# Identical Quantum Particles, Entanglement, and Individuality

**DOI:** 10.3390/e22020134

**Published:** 2020-01-23

**Authors:** Dennis Dieks

**Affiliations:** History and Philosophy of Science, Utrecht University, Princetonplein 5, 3584 CC Utrecht, The Netherlands; d.dieks@uu.nl

**Keywords:** identical quantum particles, indistinguishability, the concept of a particle, emergence, entanglement

## Abstract

Particles in classical physics are distinguishable objects, which can be picked out individually on the basis of their unique physical properties. By contrast, in the philosophy of physics, the standard view is that particles of the same kind (“identical particles”) are completely indistinguishable from each other and lack identity. This standard view is problematic: Particle indistinguishability is irreconcilable not only with the very meaning of “particle” in ordinary language and in classical physical theory, but also with how this term is actually used in the practice of present-day physics. Moreover, the indistinguishability doctrine prevents a smooth transition from quantum particles to what we normally understand by “particles” in the classical limit of quantum mechanics. Elaborating on earlier work, we here analyze the premises of the standard view and discuss an alternative that avoids these and similar problems. As it turns out, this alternative approach connects to recent discussions in quantum information theory.

## 1. Introduction

Our everyday environment is filled with distinguishable objects, which differ from each other by one or more physical characteristics. Classical physics has extended this picture into the domain of the not directly observable by the introduction of the concept of a *particle*: a classical particle is an entity characterized by an individuating set of values of physical quantities (mass, electric charge, position, momentum, etc.). It is true that two or more classical particles may have a number of physical properties in common, but they will differ at least in their spatial positions since the particles of classical theory are assumed to be impenetrable.

It is therefore possible to assign a physically defined identity to each classical particle, represented by a unique name or label. Because classical particle labels thus possess physical content (usually relating to positions and trajectories), there is no need to invoke an additional non-physical (“metaphysical”) notion of particle identity (“*haecceity*” or “primitive thisness”). This is as we expect it to be: it would be undesirable, from a methodological point of view, to introduce notions into physics that cannot be related at least in *some* way to physical quantities.

In particular, we expect a physical concept of particle identity to play a role in the dynamics. This is needed to guarantee that particles are individually addressable by empirical means—that we can pick them out by using physical interactions. In the case of classical physics, this requirement is fulfilled: by using detection techniques that are sensitive to spatial position, we can in principle identify and follow classical particles.

In the usual 6N-dimensional phase space representation of classical many-particle states, the use of physically meaningful individual labels is taken for granted. The particle with label *i* has its own axes of the total phase space associated with it, and can in principle be measured individually via interactions that make contact with only its part of phase space. It is true that, in the case of a collection of classical particles *of the same kind*, with the same intrinsic properties like rest mass and electric charge, any measuring device must couple in the same way with each particle, so that the interaction Hamiltonian has to be symmetric in the particle labels. Nevertheless, it is possible to interact with only one specific particle by using a position dependent interaction potential such that only the particle in question affects the measuring device (because all other particles are too far away). In this sense, classical particle labels are measurable quantities.

Quantum mechanics models particle systems, also if they consist of particles of the same kind, after the classical example. That is, the quantum particles are assumed to be labeled, 1,2,⋯,N, and each particle is allotted its own part of the total state space. In quantum mechanics, state spaces are Hilbert spaces and we are thus led to a tensor product structure of the total state space: $H^{N}=H_{1}⊗H_{2}⊗H_{3}⊗\cdots ⊗H_{N}$, where the *factor space* $H_{i}$ is the one-particle Hilbert space associated with the particle labeled *i*. This factor space is the analogue of the “*i*-particle part” of the classical phase space. As in the classical case, the Hamiltonian representing interactions between an external system and a collection of quantum particles of the same kind (“identical particles”) must be symmetric in the particle labels, since the interaction has the same form for all members of the collection [1].

There is nevertheless an essential difference between the quantum and classical cases. In classical physics, each single particle label is associated with a unique and complete (“pure”) one-particle state that differs from the states of all other particles. This is not so in quantum mechanics, as a consequence of the *symmetrization postulate*. This postulate ordains that the states of collections of identical particles must be symmetric or anti-symmetric under permutations of the particle labels.

For the simplest situation, namely a system consisting of two identical particles described in $H_{1}⊗H_{2}$, only states of the following form are accordingly allowed:

(1) $$|\Psi \rangle =\frac{1}{\sqrt{2}}{\{|\phi \rangle }_{1}{|\psi \rangle }_{2}\pm {|\psi \rangle }_{1}{|\phi \rangle }_{2}\}.$$

The plus sign applies to particles with integer spin (bosons) and the minus sign to particles with half-integer spin (fermions).

States of the product form $|\Psi \rangle ={|\phi \rangle }_{1}⊗{|\psi \rangle }_{2}$ are consequently forbidden in the quantum mechanics of identical particles. If states with this product form *were* allowed, we could hope to complete the analogy with classical mechanics by associating the labels 1 and 2 with the pure—and therefore maximally informative—one-particle states |ϕ〉 and |ψ〉, respectively; and in this case the labels might be empirically accessible through measurements that distinguish these two states (which would certainly be possible in the case of orthogonal states). The symmetrization postulate, however, has the consequence that both labels are *symmetrically* associated with |ϕ〉 and |ψ〉. In fact, the one-particle states derivable from (1) by partial tracing are the same in $H_{1}$ and $H_{2}$, namely 1/2{|ϕ〉〈ϕ|+|ψ〉〈ψ|}. (We here follow the custom of considering the partial traces as the *states* of the one-particle systems, since these partial traces make it possible to predict the expectation values of all observables defined in the one-particle Hilbert spaces. These partial trace states are not pure but mixed. However, they do not allow an “ignorance interpretation”: the assumption that only one pure state is actually present, but we do not know which, leads to a contradiction. For this reason, the mixed states resulting from partial tracing are often called “improper mixtures”, as they are not the results of statistically mixing pure states.) It follows that it is impossible to identify the labels 1 and 2 experimentally, on the basis of results of measurements: the partial traces associated with 1 and 2 predict identical expectation values for all observables. The “particles” labeled by factor space indices can therefore not be individually addressed by experimental methods.

The physics textbooks doctrine—which is also the received view in the field of philosophy of physics [2]—that the labels of the factor spaces in the tensor product Hilbert space of an identical particles system refer to single particles (This doctrine has been dubbed “factorism” [3].) makes it therefore impossible to distinguish these particles experimentally. This impossibility is not due to imperfections in our technical capabilities, which could be overcome at a later point in time. The lack of physical individuality of “factor space particles” is a point of principle that derives directly from the symmetrization postulate, which puts all “factorist particles” in exactly the same mixed one-particle state.

However, in circumstances in which classical physics provides an adequate description of (part of) the world, particles—in the ordinary sense of the word—do possess their own individuality based on identifying physical properties. It follows that the factorist particles represented in the factor spaces of the total Hilbert space, and labeled by the indices of these factor spaces, cannot correspond to what we ordinarily call particles: in the classical limit of quantum mechanics, the symmetrization postulate remains untouched, so that, even in this limit, factorist particles will all remain in the same state. Therefore, we need another definition of what a quantum particle is and how it is represented in the formalism if we wish to achieve a gradual transition from quantum particles to classical particles and if we want to avoid empirical inaccessibility of individual quantum particles (Section 2).

It should be noted that we do not in any way contest the *empirical* validity of the standard approach. The standard way of working with quantum mechanics is certainly able to yield correct empirical predictions that in the classical limit can be understood in terms of distinguishable classical particles. Our point is conceptual: these emerging classical particles cannot be considered to evolve smoothly, via a gradual limit transition, from underlying factorist quantum particles. We may add that in the present-day quantum physics literature (e.g., [4,5,6,7]) an awareness of the unsatisfactory character of factorism already manifests itself: in the discussion of experiments, factorism is frequently replaced by a view in which particles are associated with pure quantum states, in a vein very similar to what we are about to propose as a general strategy in this paper. See Section 5 for a brief discussion of the experiments of [4,5,6,7].

An alternative, non-factorist way of characterizing quantum particles was described in [8,9,10]. The core idea is to associate particles not with factor space labels but with *pure one-particle states* occurring in symmetrized many-particle states of the type occurring in Equation (1). Thus, instead of associating particles with the indices 1 and 2 in (1), as the standard approach does, the idea is to look for states like |ϕ〉 and |ψ〉 and to interpret these states as representing individual particles (see Section 3). This approach can immediately be seen to hold at least the promise of leading to a satisfactory classical limit: if |ϕ〉 and |ψ〉 are orthogonal, the particles represented by these states are empirically distinguishable and thus can be assigned physically grounded and empirically accessible identities, just like classical particles.

This alternative view of how quantum particles are to be represented is at variance with the official textbook doctrine and leads to new questions. One of the most significant of these concerns the notion of entanglement. According to standard definitions, a state of the form (1) represents two particles (identified by labels 1 and 2) that are entangled with each other because the state is not a product of two pure one-particle states. However, on the view that the state of Equation (1) represents one particle characterized by the state |ϕ〉 and one particle represented by |ψ〉, one may well wonder whether these particles should really be considered entangled with each other. Experience tells us that, in the case of orthogonal states, for example of particles prepared at a large distance from each other, such particles may behave in an uncorrelated way and in this case may be considered as not entangled. This connects to a two decades old discussion in the physics literature going back to work by Ghirardi et al. [11], about the definition of entanglement for identical quantum particles. This discussion has recently attracted new attention because of its relevance for quantum information theory. As we shall argue, central issues in this discussion become conceptually clearer in light of the alternative particle concept explained and defended here (Section 5).

## 2. The Standard Approach and Its Problems

Consider a situation with two identical particles that only have a non-vanishing detection probability in two non-overlapping spatial volumes, on the Left and on the Right, respectively. For the sake of simplicity, we assume in this example that the particles do not possess internal degrees of freedom, and that their total state has the following symmetrical form:

(2) $$|\Psi \rangle =\frac{1}{\sqrt{2}}{\{|L\rangle }_{1}{|R\rangle }_{2}+{|R\rangle }_{1}{|L\rangle }_{2}\}.$$

Here, the kets |L〉 and |R〉 correspond to wave functions with non-overlapping domains on the left and right, respectively. The one-particle pure states |L〉 and |R〉 occur in exactly the same way in the two factor spaces labeled by 1 and 2. As already pointed out in the Introduction, if these labels are also interpreted as particle labels, so that the state of particle 1 is represented in factor space 1 and that of particle 2 in factor space 2, we have to conclude that our two particles are in precisely the same mixed state. More generally, in the case of an (anti-)symmetrical total state of an *N*-particle system, all factor spaces contain the same one-particle states in exactly the same way so that there can be no measurable differences between the particles represented in these factor spaces.

The same conclusion can be reached formally by determining single-particle states via the procedure of taking “partial traces”: tracing out, in state (2), over the parts labeled by 2, we obtain the mixed state W=1/2{|L〉〈L|+|R〉〈R|}; and exactly the same state by tracing out over 1. According to the standard approach, these two identical mixed states are the quantum states of the two factorist particles (see footnote 1). This conclusion generalizes to (anti-)symmetric *N*-particle states, with N>2: partial tracing leads to identical results in each factor Hilbert space.

Thus, if each identical quantum particle has its own factor space and if the quantum states defined in these spaces provide complete particle descriptions, all particles in a collection of particles of the same kind must possess exactly the same physical properties. (Note that we are here referring to particle properties, if any, *before* measurement. If the quantum description by means of states is complete, identity of states entails identity of properties. What these properties are is interpretation dependent, and is not essential for this paper. In the case of a pure state, it is customary to think of the projection operator on this state as representing a possessed property; in the case of an improper mixture, one might think of *W* itself, which is an observable, as representing a non-classical “vague” property.)

The conclusion that all particles of the same sort share the same properties is extremely strange: it conflicts with the very idea of a particle. For example, since the symmetrization postulate applies to all particles of any given kind in the whole universe (See Section 5 for a further discussion and justification of this universal and global character of the symmetrization postulate.), e.g., to all existing electrons, the factorist must hold that each single electron is equally present at all positions in the universe at which there is “electron presence”. Thus, on the factorist view it would not make sense to speak about the specific electrons fired by an electron gun: all electrons in the universe equally partake in being fired by this gun. (Again, these considerations apply to factorist particles as represented in the total state, prior to measurements, and are not about the outcomes of measurements. It is well-known that local *measurement results* and their probabilities are independent of conditions at a distance. This reinforces the point that factorist particles, with their non-local character, lack immediate physical relevance: they do not enter in a natural way into the explanation of local measurement results.)

The non-local character of factorist particles and more generally their lack of individual identity are in obvious conflict with the way the notion of a particle is employed in classical physics. This is relevant because the very motivation for speaking about particles, also in quantum physics, comes from analogies with classical physics. This is clear from how the particle concept is used in actual physical practice. The Einstein–Podolsky–Rosen–Bohm experiment, much discussed in the context of Bell inequalities, furnishes a first concrete illustration of the tension between everyday physical practice and factorism. The two-electron state considered in this experiment has the form

(3) $$|\Phi \rangle =\frac{1}{\sqrt{2}}{\{|L\rangle }_{1}{|R\rangle }_{2}+{|R\rangle }_{1}{|L\rangle }_{2}{\}⊗\{|\;↑\rangle }_{1}{|↓\rangle }_{2}-{|\;↓\rangle }_{1}{|↑\rangle }_{2}\},$$

where |L〉 and |R〉 as before are states localized in non-overlapping regions on the left and right.

The standard approach now tells us that, in the situation described by the state of Equation (3) there are two particles, labeled 1 and 2, that are each in exactly the same mixed state, of which the spatial part is 1/2(|L〉〈L|+|R〉〈R|). This means that each of these factorist particles is “evenly spread out” over left and right. It follows that the way the Einstein-Podolsky-Rosen (EPR) case is commonly described, both in the foundations of physics literature and in experimental practice, namely as a situation in which there are two distinct and localized systems, one on the left and one on the right, is at odds with the official standard account in which the indices 1 and 2 are particle labels. As we shall discuss, the rejection of factorism will make it possible to interpret the state of Equation (3) as a representation of two localized systems. (However, as we shall also see, the particular form of the superposition in Equation (3) prevents an interpretation in terms of two electrons each fully characterized by a pure localized one-electron state. This will turn out to be the background of the non-locality evidenced by violations of Bell inequalities in states like that of Equation (3).)

Other examples of the mismatch between physical practice and the factorist doctrine are provided by recent experiments on particle interference. For instance, in their paper entitled “Coherence and indistinguishability of single electrons emitted by independent sources” [4], the authors (Bocquillon et al.) discuss the emission of electrons by sources that are located at a considerable distance from each other. This setup is essentially the same as that represented by the state of Equation (2), but now with a minus sign since we are dealing with fermions. According to factorism, the particles simultaneously emitted in this experiment are not initially located each at its own source, but are “distributed over both sources”. This, however, is not the way the authors conceptualize their experiment. As their title already makes clear, they think of individual electrons, each originating from one source. Such individual electrons can evidently not be of the factorist type, so the question arises of how they are represented in the formalism if not by the usual particle labels and associated mixed states. This is transparent in the paper: the authors speak of “emitters that generate a single-electron wavepacket at a well defined time”, and discuss the trajectories of these single-particle wavepackets after their emission. This clearly indicates a non-factorist conception, in which quantum particles are identified by a pure one-particle state—in the same spirit as what we will discuss in Section 3.

An additional but related reason for being dissatisfied with the factorist conception of quantum particles comes from considering the classical limit. Collections of particles of the same kind must be represented by (anti-)symmetric states by virtue of a general principle of quantum mechanics, which is not suspended when going to a classical limit situation. The sameness of partial traces in all factor spaces is consequently a generic and robust feature of quantum mechanics that survives the classical limit. This means that, even after taking this limit, all particles that were defined in the quantum formalism through reference to factor space labels still possess exactly the same properties. However, particles in the sense of the classical theory are of course individuals that are physically different from each other. Therefore, the particles that we know from classical physics cannot evolve through a gradual and smooth transition from their quantum namesakes if the latter are defined according to the factorist approach. For example, the electrons in the classical Maxwell theory of the electromagnetic field cannot be smoothly approximated by the electrons from quantum mechanics. It seems evident that this is an undesirable situation. We should expect that the quantum description continuously approximates the classical one when quantum effects become negligible. To achieve this, we have to reconsider the way in which single particles are represented in the quantum mechanics of identical particle systems.

## 3. Quantum Particles

In [3,9,10] an explicit alternative to the textbook way of using the particle concept in quantum mechanics was formulated that accords with ideas implicitly current in practice; the core motive can already be found in [8]. The central idea is to associate particles not with labels in the tensor space formalism, but instead with *pure one-particle states* that occur in the total *N*-particle state.

The motivation for this interpretative move has already been explained, but for completeness again briefly consider a state of the form

(4) $$\frac{1}{\sqrt{2}}{\{|L\rangle }_{1}{|R\rangle }_{2}-{|R\rangle }_{1}{|L\rangle }_{2}\},$$

in which |L〉 and |R〉 stand for non-overlapping wave packets at a distance from each other, on the left and right. According to the standard view, factorism, this state represents two particles that are both in the mixed state W=1/2{|L〉〈L|+|R〉〈R|}, so both equally “smeared out” over left and right. However, the nature of this state, with its two widely separated and narrow spatial regions in which something can be detected at all, suggests something else, namely that we are dealing with a situation in which there is one particle to the left and one to the right; the results of position measurements would agree with this interpretation. As noted, this interpretation is adopted without question in the actual practice of physics. In order to flesh it out, individual particles should be associated with the states |L〉 and |R〉, respectively, even though each of these states occurs in *both* factor spaces so that there is no correlation between factor space labels and individuating one-particle states.

A theoretical framework employed by Ghirardi, Marinotto and Weber [11] turns out to be useful to make these ideas more mathematically precise. Their scheme starts from the observation that, independently of the symmetrization postulate, all observables of systems of identical particles must be symmetric in the factor labels (because measurement interactions cannot distinguish between different labels, as already pointed out in the Introduction). Observables corresponding to single particle properties must accordingly be represented by symmetric projection operators (telling us whether or not the property in question is instantiated—via the eigenvalues 1 and 0 representing certainty of finding or not finding the property in question). We should therefore not consider operators of the form $P_{1}⊗I_{2}$ (with $I_{2}$ the unity operator in factor Hilbert space 2) if we want to represent empirically accessible particle properties, but rather projection operators of the form

(5) $$P_{1}⊗I_{2}+I_{1}⊗P_{2}-P_{1}⊗P_{2},$$

with *P* standing for the projection operator to be used for the relevant property in the case of a one-particle system (in which there is only one factor space). The expectation value of the operator in (5) in an (anti-)symmetric state is the probability of finding at least once the result 1 in a double *P* measurement. The final term in (5) can be omitted in the case of fermions. (The last term of the sum of projectors (5) has been added to allow for the possibility that the same one-particle state occurs twice in the total state, which may happen with bosons. In this case, the probability would become greater than 1 without the last term. In fermion states one-particle states cannot occur more than once, and in this case the last term can be omitted.)

The consistent use of symmetric projection operators makes it sometimes possible to associate a system of identical particles with definite one-particle properties represented by pure states, even though all the factor spaces contain the same mixed state. This is due to the fact that (anti-)symmetric many-particle states may be eigenstates with eigenvalue 1 of symmetric projection operators like (5), in which case it is certain that the corresponding property will be found in a measurement. For example, state (4) is an eigenstate with eigenvalue 1 of the symmetric projector $\left|L\rangle \langle L\right|⊗I_{2}+I_{1}⊗\left|L\rangle \langle L\right|$.

For such cases, Ghirardi, Marinotto, and Weber [11] suggest that the many-particle system can be thought of as built up from components each possessing a one-particle property having probability 1. Their proposal is that we thus obtain a description of an *N*-particle state in terms of *N* one-particle states although, as a point of principle, we cannot say *which* particle occupies each of these one-particle states. For example, in the case of state (4), we are entitled to say that the system consists of one particle on the left and one on the right, but it is impossible to know whether it is particle 1 or particle 2 that is located at each of these respective positions.

Our own scheme is very similar but still deviates in a subtle but conceptually significant way. As explained before, we propose to *define* and identify particles through their physical properties and to reject any notion of particle identity that is not empirically accessible. From this view, the factor space labels in the tensor product formalism cannot be taken to refer to single particles at all, since they are not measurable quantities. It is then inconsistent to associate particles with definite and measurable physical properties, and at the same time to think that they correspond to factor indices like 1 and 2. Thus, the statement that there is one particle to the left and one to the right, but that we cannot know which one is particle 1 and which one is particle 2, does not make sense according to our proposal. In our view, there is one particle to the left, characterized by |L〉, and one to the right, represented by |R〉; the indices 1 and 2 merely label factor spaces in the total product Hilbert space and are not particle names. Put differently, in our view, the factor space indices are merely formal, mathematical quantities. Admittedly, they play an important role in the definition of the total Hilbert space, but they do not possess the physical significance of particle names.

This interpretation of the factor labels as mathematical rather than physical quantities was recently challenged by Goyal, who writes [12] (pp. 8–9), in response to a similar statement in [9]:

However, such a claim leaves the challenge of formulating an alternative understanding of these indices, which, for instance, is capable of rendering intelligible the usual procedures for interpreting measurement operators. For example, if one applies the symmetrization procedure to the electrons in a helium atom, the measurement operator ${\left(x_{1}-x_{2}\right)}^{2}$ is ordinarily interpreted as representing a measurement of the squared-distance between the two electrons; but it is unclear how one would justify such an interpretation if the indices 1 and 2 have a “merely formal significance”.

Consider, in order to respond to this objection, the result of applying the observable ${\left(x_{1}⊗I_{2}-I_{1}⊗x_{2}\right)}^{2}$ to state (4). Straightforward calculation yields:

(6) $${\left(x_{1}⊗I_{2}-I_{1}⊗x_{2}\right)}^{2}\frac{1}{\sqrt{2}}{\{|L\rangle }_{1}{|R\rangle }_{2}-{|R\rangle }_{1}{|L\rangle }_{2}\}={\left(l-r\right)}^{2}\frac{1}{\sqrt{2}}{\{|L\rangle }_{1}{|R\rangle }_{2}-{|R\rangle }_{1}{|L\rangle }_{2}\}.$$

State (4) is therefore an eigenstate of ${\left(x_{1}⊗I_{2}-I_{1}⊗x_{2}\right)}^{2}$ with eigenvalue ${\left(l-r\right)}^{2}$, with *l* denoting the coordinate at which |L〉 is centered and *r* the position of |R〉. The quantity ${\left(l-r\right)}^{2}$ is therefore the squared distance between our two non-factorist particles defined and labeled by *L* and *R*, respectively. In other words, the observable ${\left(x_{1}⊗I_{2}-I_{1}⊗x_{2}\right)}^{2}$ represents a physically meaningful squared mutual particle distance; and it does so without invoking any other meaning of 1 and 2 than the mathematical one of indices of the factor spaces in the tensor product space—there is no need to think here of 1 and 2 occurring in the expression for the observable as particle names. Is it intelligible why the distance between non-factorist particles can be expressed this way? Certainly: this is because |L〉 and |R〉 in state (4) occur in *different* factor spaces, either in the order 1−2 or 2−1, so that $\left(x_{1}⊗I_{2}-I_{1}⊗x_{2}\right)$ picks out the difference in position between the Left and Right particle. That *both* orders appear in the state (4) illustrates once again that 1 and 2 themselves do not correlate to either *L* or *R*, so that they cannot function as unambiguous particle names.

The formalism of Ghirardi et al. [11] is independent of this conceptual issue. Their argument demonstrates how it is possible, and why it is reasonable, to associate *N* pure one-particle states with an (anti-)symmetric *N*-particle state if this total (anti-)symmetric state can be obtained from symmetrizing or anti-symmetrizing an *N*-fold product state. Such (anti-)symmetrized product states are always eigenvectors of symmetric projection operators of the form (5) or its generalizations to *N*-fold products. In the case of (anti-)symmetrized product states, we can therefore always find *N* one-particle states that define one-particle subsystems.

In the case of anti-symmetrized product states (fermions), we can always find *mutually orthogonal* single-particle states with the help of the above recipe. These states are fully distinguishable by empirical means. In the case of bosons, this mutual orthogonality of states is not guaranteed. Accordingly, fermionic states that are anti-symmetrized product states can always be interpreted in terms of fully distinguishable particles.

Bosonic symmetrized product states do not always allow such a distinguishable particle description, however, because the one-particle states occurring in them may overlap and even coincide. In such situations, bosons are better described as assemblies of field quanta (in a Fock space occupation number representation—see [9,10,13]) than as individual particles. There is therefore a basic difference between fermions and bosons, which can be expressed by means of the Pauli exclusion principle: no two fermions can be in the same state. Note, however, that this common formulation of the exclusion principle is in conflict with the official standard view that particles correspond to factor spaces: the reduced particle states in all factor spaces are identical, even for fermions. In the alternative scheme explained here, the Pauli exclusion principle *can* be maintained in its usual form, though. (It should be noted, nevertheless, that this fundamental difference between fermions and bosons does not entail that empirical (sampling) probability distributions should always be different for fermionic and bosonic systems; see [14].)

In a field picture of bosons in the same state the number *N* should not be seen as the result of counting *N* individual entities, but rather as a mass noun referring to a total undifferentiated quantity. Think of the analogy with a total quantity of *N* liters of a liquid, which does not consist of *N* well-defined individual liter-entities [13].

Not only does this way of implementing the quantum particle concept fit the actual practice of physics, but it also evades the objection that it cannot reproduce what we expect from the transition to the classical limit, namely quantum particles that gradually transform into their classical counterparts by becoming more localized and by following approximately classical trajectories. Of course, narrow quantum wave packets disperse quickly, and will only move according to approximately classical trajectories for short periods of time. In order to arrive at the correct classical limit, conditions must therefore be fulfilled that counteract the consequences of dispersion. Decoherence, which can be thought of as the effect of frequent successive position measurements by the environment, must be assumed to play a vital role here, in addition to the usual conditions of Ehrenfest’s theorem.

To sum up, what we propose is the definition and identification of quantum particles by distinctive physical properties, represented by one-particle projection operators and their eigenstates. The thus defined quantum particles can be labeled on the basis of their individuating physical characteristics. These physical labels do not coincide with the factor indices occurring in the total quantum state—the latter remain associated in the same way with *all* pure one-particle states even in the classical limit, and therefore cannot refer to individual particles. It should be mentioned at this point that the decomposition in terms of such states as given in Equation (1) is not unique. The equality of the coefficients appearing in front of the terms in the (anti-)symmetric superposition is responsible for a degeneracy that allows infinitely many alternative decompositions, in addition to the one in terms of |L〉 and |R〉. Thus, the set of properties that distinguish the quantum particles is underdetermined by the procedure as we have outlined it. To make the definition of the particles unique, some additional ingredient is needed, which picks out a privileged particle–properties basis. It is plausible, and in accordance with the classical picture of particles as localized entities, to take the position basis as privileged [9]. This ties in with the argument, to be discussed in Section 5, that the possibility of interpreting a total state as a representation of several individual particles does not only depend on the form of the state, but also depends on the form of the interactions with the environment. Measurement interactions used to verify the presence of particles have a local character, and this endows “position” with a privileged status (see [15] for a tentative exploration of this idea).

## 4. Particles without Factor Labels

The irrelevance of factor space indices for the identification of individual particles in a collection of identical particles raises the question of whether it is possible to describe these particles without appealing to non-empirical labels at all. It seems clear from the outset that the answer to this must be affirmative because a similar treatment already exists in quantum field theory. The formalism of second quantization deals with *field quanta* as entities that are only identifiable through the states (modes) they are in and that do not carry individual labels. A formalism in the same spirit, but now from the outset meant for *particles* and without the formalism of creation and annihilation operators, was introduced by Lo Franco et al. [16]; see also [17,18,19].

The core idea is to introduce a new many-particle Hilbert space (corresponding to the (anti-)symmetrical sector of the usual tensor product space) spanned by vectors of the form |α,β〉, which represent one particle characterized by the one-particle state |α〉 and one in the state |β〉. The state of Equation (4) would in this formalism be written as |L,R〉, with the interpretation that there is one particle on the left-hand side (*L*), the other on the right-hand side (*R*). As in the previous section, we here deviate slightly but from a conceptual viewpoint significantly from the literature [16,18,19] in that we *define* our particles by means of the characteristics *L* and *R*. As a result, these particles are physically identifiable entities and their “labels” *L* and *R* are measurable quantities. By contrast, Lo Franco et al. [16] introduce their formalism by stating: “Indistinguishability requires that the identical particles cannot be individually addressed”, which is still based on the idea the factor labels refer to single particles. This leads to the confusing notion that we have a set of distinguishable one-particle states, but cannot know *which* particle is in any given state. As explained in Section 3, on our view it makes no sense to ask for the label of the particle in any given state.

To fix the properties of the new two-particle Hilbert space, we have to equip it with an inner product. This can be done in the following way [16,17]:

(7) $$\langle \alpha^{\prime },\beta^{\prime }|\alpha ,\beta \rangle :=\langle \alpha^{\prime }|\alpha \rangle \langle \beta^{\prime }|\beta \rangle +\eta \langle \alpha^{\prime }|\beta \rangle \langle \beta^{\prime }|\alpha \rangle ,$$

where η=±1 and where 〈ϕ|ψ〉 is the one-particle inner product of the standard formalism.

The two possible values of η, −1 and +1, correspond to the difference between fermions and bosons. It follows from (7) that the order of α and β in the state |α,β〉 is not without significance: |α,β〉=η|β,α〉, so that exchanging one-particle states in the total state leads to a global phase shift in the case of fermions.

The set of state vectors of the form |α,β〉 spans a two-particle Hilbert space that is isomorphic to the (anti-)symmetric subspace of the tensor product space H⊗H, but does not possess a tensor product structure itself. This suggests that, in general, states like |α,β〉, even though they can be interpreted as instantaneously representing one individual particle in state |α〉 and one in state |β〉, do not represent a system of two objects each having its own dynamical evolution in its own state space. Indeed, the form of the inner product of Equation (7) shows that a one-particle state at an instant $t^{\prime }$ may contain contributions from both one-particle states at an earlier instant *t*, $t^{\prime }>t$. The classical notion of a particle that retains its identity over time by following a well-defined trajectory need therefore not be applicable in quantum mechanics, even if we start with well-defined and empirically discernible particle states. (It is interesting to compare our argument here, which takes its starting point in the formalism of quantum mechanics, with the analysis in [12], which starts from an operational point of view and proposes that interpretations of empirical data with and without “genidentical” particles (i.e., particles keeping their identity by following trajectories) should be seen as complementary. We save a discussion of this alternate viewpoint for another occasion.) However, it is also visible from Equation (7) that there is the possibility of limiting situations in which identity over time does become an applicable concept. This happens when the second term on the right-hand side of Equation (7) vanishes because $\langle \alpha^{\prime }|\beta \rangle$ and $\langle \beta^{\prime }|\alpha \rangle$ are both zero (think here of $|\alpha^{\prime },\beta^{\prime }\rangle$ as the time-evolved version of |α,β〉). In this case, the ordinary tensor product form of the inner product is recovered, so that we are entitled to represent |α〉 and |β〉 as vectors in two different factor spaces in a tensor product space. This shows once again, at least in principle, that the classical particle concept has the possibility of emerging from its quantum counterpart if we define quantum particles through their states.

## 5. Particles and Entanglement

Classical particles possess their properties autonomously, independently of whether or not there are other particles. Of course, particle properties will generally change over time, as a result of interactions with other particles and fields. However, this dynamical picture only makes sense on the very assumption that each particle can be assigned its own properties in the first place. According to classical physics, these particle properties, which exist independently of whether or not any measurements are in fact undertaken, are sufficient to explain the outcomes of measurements on particle systems.

The general situation in quantum mechanics is different. Many-particle systems may be in entangled states, and in this case measurement results and the correlations between them cannot be fully accounted in terms of preexisting values of single-particle quantities. In these circumstances, the notion itself of a single particle as an autonomous individual becomes problematic because entangled “*N*-particle systems” cannot be thought of as consisting of *N* independently existing components whose individual properties determine the properties of the total system. (If one assumes the existence of instantaneous many-body interactions, as in the Bohm theory, a different analysis applies. Our argument here is meant to be understood within standard quantum mechanics.)

In quantum mechanics, the notion of a particle can therefore not be as fundamental as it was in classical physics (see Section 6). Only if there are no observable effects of entanglement can the classical particle concept become applicable. This raises the question of whether states of the form |α,β〉, in the formalism of the previous section, are entangled or not. The way we have discussed these states, in terms of one particle fully characterized by |α〉 and one by |β〉, suggests the absence of entanglement. This appears plausible: we could, for example, be dealing with two particles that have never interacted and find themselves at a great distance from each other, so that there seems no reason to expect any correlations between them. However, in the standard formalism, the corresponding two-particle state is $\frac{1}{\sqrt{2}}\{{|\alpha \rangle }_{1}{|\beta \rangle }_{2}\pm {|\beta \rangle }_{1}{|\alpha \rangle }_{2}\}$, which is not a product state and therefore does suggest the presence of entanglement.

This issue was first addressed by Ghirardi, Marinotto, and Weber in their seminal paper [11]. They argued (in line with our discussion in Section 1 and Section 2) that (anti-)symmetrized product states of identical particles, like $\frac{1}{\sqrt{2}}\{{|\alpha \rangle }_{1}{|\beta \rangle }_{2}\pm {|\beta \rangle }_{1}{|\alpha \rangle }_{2}\}$, should be considered as *not* entangled. However, this conclusion needs to be qualified, as we will now see.

An essential premise in the argument by Ghirardi et al. is that the observables that are measured on a system probe individual one-particle properties and do not mix one-particle states like |α〉 and |β〉. That is, the measured observables are assumed to be such that they have non-vanishing expectation values either in |α〉 or in |β〉, but not in both. This assumption is valid for the observables normally considered in EPR–Bohm type of experiments: these are of the form $P_{L}.\sigma_{n}⊗P_{R}.\sigma_{n^{\prime }}+P_{R}.\sigma_{n^{\prime }}⊗P_{L}.\sigma_{n}$, with $P_{L}=\left|L\rangle \langle L\right|$ and $P_{R}=\left|R\rangle \langle R\right|$ the projector operators on the non-overlapping wave packets on the left and right, respectively. When we now consider an anti-symmetrized product state of the form

(8) $$\frac{1}{\sqrt{2}}{\{|L\rangle }_{1}{|R\rangle }_{2}{|\;↑\rangle }_{1}{|\;↓\rangle }_{2}\;-{|R\rangle }_{1}{|L\rangle }_{2}{|\;↓\rangle }_{1}{|\;↑\rangle }_{2}\},$$

it is easy to see that all results of measurements in this state are identical to the results of measuring $P_{L}.\sigma_{n}⊗P_{R}.\sigma_{n^{\prime }}$ in the product state ${|L\rangle }_{1}{|R\rangle }_{2}{|\;↑\rangle }_{1}{|\;↓\rangle }_{2}$. This entails that Bell inequalities will not be violated: under the mentioned measurement conditions (8) is effectively equivalent to a product state. By contrast, measuring the same symmetrical observables in the EPR–Bohm state (3) will result in the appearance of cross terms in the expectation values, which is responsible for the fact that, in that state, Bell inequalities *are* violated.

This result that (anti-)symmetrized products of states are effectively equivalent to product states, clearly depends on the assumed restriction on the class of observables that we were allowed to measure. This restriction only permits observables whose expectation values will not show interference between the states of the two particles (defined by the states |α〉 and |β〉 in |α,β〉).

In view of this limitation, we have to adapt our earlier statements suggesting that (anti-)symmetrized product states like (8) represent a collection of autonomous single particles *tout court*: this interpretation can only be maintained with the proviso that observables that mix one-particle states will not be considered.

In classical physics, individual particles differ at least in their positions: they are small, local entities that cannot overlap spatially. This motivates the use of the notion of individual quantum particles especially in cases in which the relevant one-particle states are (almost) non-overlapping in space. If one-particle states *do* overlap, this will show up in local measurements taking place at a distance from each other because there will be effects of entanglement [20] that signal the breakdown of the localized particle concept. An extreme example is the case in which a two-electron state is built up from two single-electron states with the *same* spatial part |ψ〉:

(9) $$\frac{1}{\sqrt{2}}{|\psi \rangle }_{1}{|\psi \rangle }_{2}{\{|\;↑\rangle }_{1}{|\;↓\rangle }_{2}\;-{|\;↓\rangle }_{1}{|\;↑\rangle }_{2}\}.$$

Local measurements performed at *L* and *R*, both within the region where ${\left||\psi \rangle \right|}^{2}>0$, will project this state into an effective state that has exactly the form of the Einstein-Podolsky-Rosen-Bohm state (3):

(10) $$|\Phi \rangle =\frac{1}{\sqrt{2}}{\{|L\rangle }_{1}{|R\rangle }_{2}+{|R\rangle }_{1}{|L\rangle }_{2}{\}⊗\{|\;↑\rangle }_{1}{|↓\rangle }_{2}-{|\;↓\rangle }_{1}{|↑\rangle }_{2}\}$$

and will therefore violate Bell inequalities in spin measurements performed at *L* and *R*.

Localization is thus a necessary condition to come close to a classical particle picture. However, even if this condition is satisfied, we have to make the additional requirement that only *local one-particle observables* are measured, in order to avoid interference between one-particle states. If the latter condition is not fulfilled, the results of measurements on (anti-)symmetrized product states like (8) may again violate Bell inequalities, even when the wave packets belonging to |L〉 and |R〉 are very far apart from each other in space. At first sight, non-local measurements bringing out interference between such spatially separated states might appear difficult to realize because interactions are typically governed by local field theories with potentials that are position dependent. However, the effects of non-local measurements can be produced by combinations of local interactions, as shown by experiments of the Hong–Ou–Mandel type [5,6,7,21]. For two electrons, the core idea of this experiment translates into the following.

Suppose that we have two electron guns, one to the Left and one to the Right, and suppose that each of these devices fires exactly one electron—one with spin up in the *z*-direction, the other with spin down. (We here follow the way in which experiments of this kind are commonly described in the physics literature. As pointed out in Section 2, this description identifies particles with one-particle states and so violates factorism.) Since the two electrons are identical fermions, we have to anti-symmetrize the total wave function, so that the total state has the form of (8). The two electron wave packets will evolve independently. Suppose that, after some time, each of them is split by a beam splitter, in a local process; and that this happens in such a way that, of both original packets, one half is directed to the location $L^{\prime }$ and the other half to the location $R^{\prime }$.

In detail, suppose that the time evolution can be represented as follows:

(11) $$\begin{array}{ccc} |L\rangle & \to & \frac{1}{\sqrt{2}}\left(|L^{\prime }\rangle +|R^{\prime }\rangle \right), \end{array}$$

(12) $$\begin{array}{ccc} |R\rangle & \to & \frac{1}{\sqrt{2}}\left(|L^{\prime }\rangle -|R^{\prime }\rangle \right), \end{array}$$

where $|L^{\prime }\rangle$ and $|R^{\prime }\rangle$ correspond to wave packets localized at $L^{\prime }$ and $R^{\prime }$, respectively.

After the evolution, the total state is still an anti-symmetrized product state: if we call the states into which |L〉 and |R〉 have evolved ϕ and ψ, respectively, the final total state can be written as

(13) $$\frac{1}{\sqrt{2}}{\{|\phi \rangle }_{1}{|\psi \rangle }_{2}{|\;↑\rangle }_{1}{|\;↓\rangle }_{2}\;-{|\psi \rangle }_{1}{|\phi \rangle }_{2}{|\;↓\rangle }_{1}{|\;↑\rangle }_{2}\}.$$

According to the criterion formulated by Ghirardi, Marinotto, and Weber ([11], see also [22]), and our discussion in Section 1 and Section 2, this state represents two independent particles, characterized by |ϕ〉|↑〉 and |ψ〉|↓〉, respectively, for which we would not expect violations of Bell inequalities. This particle interpretation and the expectation that Bell inequalities are respected will be confirmed if we detect and identify the particles by performing measurements of |ϕ〉〈ϕ| and |ψ〉〈ψ|.

However, if we perform *local* measurements, by using electron detectors positioned at $L^{\prime }$ and $R^{\prime }$, an interpretation of the results in terms of independent particles becomes problematic, in spite of the fact that both initial one-particle states were localized far apart from each other and evolved independently and unitarily. This is so because each of the two initial particles can be found both at $L^{\prime }$ and at $R^{\prime }$. This circumstance is responsible for the appearance of correlations between measurement results that can only be explained by entanglement [21]. (The possibility of verifying interference between initially widely separated wave packets, in an (anti-)symmetric total state, demonstrates that the validity of the symmetrization postulate for the total state of widely separated particles of the same kind can be empirically tested. It is sometimes suggested that the symmetrization postulate needs not be respected for particles at a great distance, but this is only a pragmatic rule of thumb that simplifies calculations in the case of local interactions without any mixing of states.)

When in a series of repetitions of the experiment we post-select the measurements in which one electron is found at $L^{\prime }$ and one at $R^{\prime }$, correlations between the particle spins found at the two different locations can be computed from the components of state (13) that have the spatial parts $|L^{\prime }\rangle_{1}{|R^{\prime }\rangle }_{2}$ or $|R^{\prime }\rangle_{1}{|L^{\prime }\rangle }_{2}$. We thus find the effective state

(14) $$|\Phi \rangle =\frac{1}{2}{\{|L^{\prime }\rangle }_{1}|R^{\prime }\rangle_{2}-|R^{\prime }\rangle_{1}|L^{\prime }\rangle_{2}{\}⊗\{|\;↑\rangle }_{1}{|↓\rangle }_{2}+{|\;↓\rangle }_{1}{|↑\rangle }_{2}\},$$

which is not an anti-symmetrized product: it is the superposition of *two* anti-symmetrized product states (a superposition of two “Slater determinants”). This qualifies it as an *entangled* two-electron state. Therefore, if we post-select experiments in which both detectors fire, we find correlations between spin measurement outcomes that cannot be explained locally on the basis of preexisting values. These correlations are able to violate Bell inequalities. The spin part of state (14) is of course different from the EPR–Bohm singlet state; it is not rotation invariant. The correlation between spin measurements predicted by this state in the directions α and β (with α and β the angles with respect to the polarization direction *z*) is sinα.sinβ−cosα.cosβ. It is simple to find directions such that this correlation function produces violations of the CHSH inequality.

The existence of entanglement of this kind (“measurement-induced entanglement”) has been established as an important tool in experimental practice and has become a significant resource in quantum information research (see, e.g., [20,23,24,25,26]).

## 6. Classical Particles as *Emergent* Entities

In Section 3, we have explained and defended the interpretation of (anti-)symmetrized products of one-particle states as representations of particles that are characterized by the one-particle states from which the total state is constructed. However, the argument from the previous section shows that, although this proposal evades many of the problems of factorism, it still does not lead to descriptions that fully agree with what we expect from a particle picture. In particular, the quantum mechanics of identical particle systems always leaves open the possibility of interference between particle states and the violation of Bell inequalities in measurements of certain observables. Therefore, in order to come close to a classical situation, a number of conditions should be fulfilled: we should make sure that the single-particle states are (approximately) localized and importantly that “measurement-induced entanglement” is avoided. In other words, the classical particle picture will only become (approximately) applicable under special circumstances and can only represent a fringe area of the quantum world.

As a rule, so-called “many-particle quantum states” will not admit a plausible particle interpretation, since most of them are not (anti-)symmetrized product states. The EPR–Bohm state (3) already illustrates this: although its spin part ${\{|\;↑\rangle }_{1}{|\;↓\rangle }_{2}-{|\;↓\rangle }_{1}{|\;↑\rangle }_{2}\}$ is an anti-symmetrized product, this does not hold for the complete state, which is therefore entangled. This entanglement is responsible for the non-factorizability of joint probabilities of measurement outcomes on the two wings of a Bell experiment, and consequently for violations of Bell inequalities. In order to have factorizable probabilities for measurements on the two wings of the experiment, and, in order to be consistent with the picture of two independent particles with complete sets of definite one-particle properties, we should rather have a state like (8), which *is* an anti-symmetrized product of localized one-particle states. In this state, there will be no violations of Bell inequalities and therefore no no-go results for local classical models, on the condition that we confine ourselves to local measurement scenarios. In the usual Alice–Bob scheme, state (8) can thus be taken to represent a situation in which there are two particles, one on the left and one on the right, possessing spin up and spin down, respectively. The important difference with the EPR–Bohm case is that in (8) the locations *L* and *R* are correlated with definite spin eigenstates, whereas in (3) the spin part is independent of location and thus represents a global—non-particulate—property of the system.

States like (8) therefore come close to representing a classical situation, in which particles can be labeled by their properties: in this case L,↑ and R,↓, respectively (note that this labeling is non-factorist, since these particle labels differ from the factor space labels 1 and 2). Even so, as we have seen, results of measurements will only accord with a classical particle picture when measurements do not mix the two particle states. As we have noted in Section 5, it is possible to realize evolutions in which states are mixed by using specific combinations of local interactions. In situations where this happens, a particle picture is again not adequate.

Quantum states generally manifest features of holism and non-locality, and it is therefore only in special cases that the notion of a global system built up from individual and local components becomes valid. Classical particles therefore *emerge* [9,10] under quite specific conditions from the world described by quantum mechanics.

Evidently, the details of the physical mechanisms that are responsible for washing out the effects of entanglement and for making the concept of a localized particle applicable need a separate and more precise analysis. In anticipation of such an analysis, it seems clear that in many everyday situations frequent local interactions with the environment will lead to quick and efficient decoherence, and as a result to effective collapses into (almost) localized particle states. In addition, the locality of ordinary measurement interactions is important: local measurements will not mix localized states unless they are combined in special setups like those discussed in Section 5.

## 7. Discussion

In the world of everyday experience, individual localized objects constitute an essential part of “what there is”. Classical physics has generalized this everyday conception of the world by introducing the concept of a particle as a basic category. These classical particles are individuated by their properties: each single particle has a unique set of physical characteristics. At first sight, this picture seems completely irreconcilable with the quantum mechanics of systems of “identical particles”: because of the symmetrization postulate, all identical quantum particles appear to be in the same physical state and therefore seem to lack all individuality. However, as we have argued, this conclusion depends on the adoption of “factorism”, the doctrine that the labels of the factor spaces in the tensor product Hilbert space refer to single particles. In spite of the fact that factorism is part of standard wisdom, it is a physically unreasonable doctrine. Moreover, there is a plausible alternative, namely the identification of particles with the help of one-particle states that occur in the total many-particle state.

This alternative holds the prospect of leading to the right classical limit, in which quantum particles come close to the behavior of classical particles. It is also in accordance with how the particle concept is used in the practice of physics, even in present-day high-energy physics.

The alternative approach also connects to recent work in quantum information theory. It is essential for quantum information theory to have a robust notion of entanglement, since entangled systems constitute the instrument with which to achieve non-classical information transfer. The usual way of defining entanglement is to say that any state that is not a product of one-particle states is entangled. Because of the symmetrization rules, this definition has the consequence that all many-particle states of particles of the same kind (identical particles) are entangled. However, this conclusion is challenged by the fact that systems described by such states can behave as consisting of independent and individual particles, for which standard quantum information protocols do not work. The identification of particles with one-particle states instead of factor labels makes it possible to dissolve part of this problem, by making a distinction between physically spurious entanglement between “*factor particles*” and genuine entanglement between *physical* particles. Even so, it must be taken into account that all qualifications of systems as consisting of independent component particles should be seen as relative to a restricted class of interactions with which the system is to be probed.

In general, the many-particle states of identical particles allowed by quantum mechanics are not (anti-)symmetrized products. In such states, entanglement is manifest, and accordingly it then is not natural to interpret the total system as built up from individual entities each characterized by its own one-particle state. Such manifestly entangled states are exactly the states that are responsible for the typical quantum features of non-local correlations and violations of Bell inequalities. This illustrates the trade-off between the applicability of the notion of a particle on the one hand and the occurrence of typical quantum effects on the other. It is precisely when holism, non-locality, and entanglement disappear from sight that the classical world and its particles emerge. By contrast, on the fundamental level of quantum mechanics, there is overwhelming evidence that the concept of global systems as being composed of autonomous individuals is not theoretically fruitful.

Awareness of how particles can and should be represented in quantum mechanics, of why the quantum world in its basic features is non-particulate, and of how features of the classical world in principle emerge from the quantum world, provides useful conceptual resources for the study of quantum processes. Phenomena like violations of Bell inequalities and non-classical information transfer become better understandable when we realize that the systems involved do not consist of particles in the usual sense; that, for example, we should not speak and think of Alice’s and Bob’s particles with their individually possessed spins. Typically, in such situations, there are (approximately) localized states that are *not* correlated with definite spin states, so that the full applicability of the notion of a particle defined by a complete one-particle state fails. Adjusting our explanations better to these features of the quantum formalism may enable a better understanding of what is going on in the quantum world (see for attempts in this direction [15,27]).
